# Supplementary material for: How Bank Vole-PUUV Interactions Influence the Eco-Evolutionary Processes Driving Nephropathia Epidemica Epidemiology—An Experimental and Genomic Approach
Source: Pathogens. 2020 Sep 25;9(10):789. doi: 10.3390/pathogens9100789 (PMC7599775; doi:10.3390/pathogens9100789)
Supplement: Supplementary file 1 [file pathogens-09-00789-s001.zip › Supplementary table S1.docx]

**Supplementary table S1**: Generalized linear mixed models (GLMMs) results testing the effect of the status of infection (PBS or virus), PUUV strain, bank vole population and *‘time’* on bank vole’s weight. Significant *p*-value are in bold.

| Responses variables | Fixed effects | Df | *X^2^* | *p*-value |
| --- | --- | --- | --- | --- |
| Weight | Status of infection | 1 | 0.06 | 8.11 x 10^-1^ |
|  | PUUV strain | 1 | 2.7 x 10^-3^ | 9.58 x 10^-1^ |
|  | Bank vole population | 1 | 1.75 | 1.86 x 10^-1^ |
|  | Time | 5 | 50.65 | **1.02 x 10^-9^** |
