# Supplementary material for: How Bank Vole-PUUV Interactions Influence the Eco-Evolutionary Processes Driving Nephropathia Epidemica Epidemiology—An Experimental and Genomic Approach
Source: Pathogens. 2020 Sep 25;9(10):789. doi: 10.3390/pathogens9100789 (PMC7599775; doi:10.3390/pathogens9100789)
Supplement: Supplementary file 1 [file pathogens-09-00789-s001.zip › Supplementary table S4.docx]

**Supplementary table S4**: Mean and standard deviation of PUUV viral loads for each PUUV strain. bank vole population and *‘time’* (day post infection). and detected in the lungs. liver. salivary gland and rectum. Dpi = day post-infection. Generalized linear model: ns = non significant, * indicates *p*-value < 0.05.

| Samples | Fixed effects |  | PUUV viral load mean  (RNA copy/mg (log10)) | PUUV viral load standard deviation |
| --- | --- | --- | --- | --- |
| Lungs | PUUV strain ^ns^ | Hargnies | 5.51 | 2.55 |
|  |  | Vouzon | 5.30 | 1.57 |
|  | Bank vole population ^ns^ | Ardennes | 5.70 | 2.25 |
|  |  | Loiret | 5.07 | 1.86 |
|  | **Time ^*^** | 3 dpi | **4.42** | **2.21** |
|  |  | 7 dpi | **7.37** | **1.15** |
|  |  | 14 dpi | 5.69 | 1.43 |
|  |  | 21 dpi | **5.32** | **0.58** |
|  |  | 28 dpi | **3.84** | **2.59** |
| Liver | PUUV strain ^ns^ | Hargnies | 3.45 | 3.08 |
|  |  | Vouzon | 3.20 | 2.54 |
|  | Bank vole population ^ns^ | Ardennes | 3.74 | 2.76 |
|  |  | Loiret | 2.86 | 2.80 |
|  | **Time ^*^** | 3 dpi | **3.98** | **1.96** |
|  |  | 7 dpi | **6.26** | **1.14** |
|  |  | 14 dpi | **1.46** | **2.68** |
|  |  | 21 dpi | **1.80** | **2.49** |
|  |  | 28 dpi | **2.37** | **2.54** |
| Salivary glands | PUUV strain ^ns^ | Hargnies | 4.07 | 1.75 |
|  |  | Vouzon | 2.38 | 2.46 |
|  | Bank vole population ^ns^ | Ardennes | 3.39 | 2.18 |
|  |  | Loiret | 2.98 | 2.43 |
|  | **Time ^*^** | 3 dpi | **1.04** | **1.88** |
|  |  | 7 dpi | **4.87** | **1.73** |
|  |  | 14 dpi | 2.85 | 2.36 |
|  |  | 21 dpi | **4.60** | **0.43** |
|  |  | 28 dpi | 3.18 | 2.00 |
| Rectum | PUUV strain ^ns^ | Hargnies | 1.28 | 2.28 |
|  |  | Vouzon | 2.01 | 2.42 |
|  | Bank vole population ^ns^ | Ardennes | 2.00 | 2.41 |
|  |  | Loiret | 1.29 | 2.29 |
|  | **Time ^*^** | 3 dpi | **0.00** | **0.00** |
|  |  | 7 dpi | **3.23** | **2.60** |
|  |  | 14 dpi | 1.41 | 2.43 |
|  |  | 21 dpi | **2.86** | **2.40** |
|  |  | 28 dpi | 1.13 | 2.09 |
