# Supplementary material for: How Bank Vole-PUUV Interactions Influence the Eco-Evolutionary Processes Driving Nephropathia Epidemica Epidemiology—An Experimental and Genomic Approach
Source: Pathogens. 2020 Sep 25;9(10):789. doi: 10.3390/pathogens9100789 (PMC7599775; doi:10.3390/pathogens9100789)
Supplement: Supplementary file 1 [file pathogens-09-00789-s001.zip › Supplementary table S5.docx]

**Supplementary Table S5** : Selected sequencing samples and definition of the majors SNPs.

| **Strains** | **Bank voles** | **Individuals** | **Dpi** | **Samples** | **AA of majority variant** | **AA position** |
| --- | --- | --- | --- | --- | --- | --- |
| **Hargnies** |  |  |  | Cell culture | Gln^1^ (Q) | 63 |
| **Hargnies** | Ardennes | 17.45.11 | 14 | Lungs | Arg^2^ (R) | 63 |
|  |  |  |  | Liver | Arg^2^ (R) | 63 |
|  |  |  |  | Salivary glands | Arg^2^ (R) | 63 |
|  |  |  |  | Bladder | Arg^2^ (R) | 63 |
|  |  |  |  | Rectum | Arg^2^ (R) | 63 |
| **Vouzon** |  |  |  | Cell culture | Ser^3^ (S) | 28 |
| **Vouzon** | Loiret | 18.99.J10 | 14 | Lungs | Ser^3^ (S) | 28 |
|  |  |  |  | Liver | - | - |
|  |  |  |  | Salivary glands | Ser^3^ (S) | 28 |
|  |  |  |  | Rectum | Ser^3^ (S) | 28 |

^1^Gln : Glutamine ; ^2^Arg : Arginine ; ^3^Ser : Serine
