# Supplementary material for: How Bank Vole-PUUV Interactions Influence the Eco-Evolutionary Processes Driving Nephropathia Epidemica Epidemiology—An Experimental and Genomic Approach
Source: Pathogens. 2020 Sep 25;9(10):789. doi: 10.3390/pathogens9100789 (PMC7599775; doi:10.3390/pathogens9100789)
Supplement: Supplementary file 1 [file pathogens-09-00789-s001.zip › Supplementary table S7.docx]

**Supplementary table S7**: GLMs results testing the effect of NE cross-infections and organs tested on the mean of complexity percent. Significant *p*-value are in bold.

| Responses variables | Fixed effects | Df | *F* | *p*-value |  |
| --- | --- | --- | --- | --- | --- |
| Complexity percent | Cross-infections | 1 | 68.83 | **8.94 x 10^-7^** |  |
|  | Organs | 2 | 3.91 | **4.46 x 10^-2^** |  |
